# Moral Sanitary Economy
*"Moral Sanitary Economy." By Henry M'Cormack, M.D., Consulting Physician to the Belfast General Hospital, &c. &c. "Education, Health, Order, Competence." Belfast, 1853, pp. 150.


**Published:** 1853-10-01

**Authors:** 


### Art. II—MORAL SANITARY ECONOMY.*

We are living in one of those epochs which constitute a crisis in the history of the human race. The character of the entire world—mind, manners and habits of life—are undergoing great changes. May we not ask whether the present generation will be in a position to recognise their ancestors before the expiration of another thirty years? The remarkable revolutions of antiquity, if more formidable, were not less momentous than the present. The dispersion at Babel, the military migrations of Sesostris, and the armed hordes who overthrew Media, Lydia, Syria, Assyria, and Egypt, in the lifetime of a single individual, the far-famed Cyrus of antiquity, are but transient events when compared with what is happening before our eyes. They swept like a torrent across the civilized portions of the globe, but as quickly subsided, and commingled with those whom they had so suddenly and violently overwhelmed. An amalgamation was formed between the conquerors and the conquered, the nations recovered from the shock, and the world renewed its course with apparently but little interruption. Even the inroads of the northern barbarians under Alaric, Attila, and Genseric, were not so much the cause of the fall of Rome, as they were, in a great measure, the result of a declining policy in the government of the mistress of the world. The thirty legions, whose arms had extended the limits of the empire at the command of Pompey, Cæsar, and Vespasian, disappeared beneath the sceptre of their feeble successors; the frontiers had been left unguarded, and the rapacious invaders descended without opposition on the inviting spoil. The world was destitute of a governing head. The masses were illiterate, rude, and brutal. The earth withered wherever they trod. The way was open for the ruthless Saracens to pounce upon the wreck of ages. They devoured, as they advanced along, the provinces of Africa from Suez to Gades. They crossed the Straits, landed upon Spain, surmounted the Pyrenees, and alighted like locusts at the gates of Poictiers, from whence they were driven back by the mail-clad warriors of the north. The social confusion of the middle ages ensued, until mankind were awakened from their enthusiastic dream of chivalry and devotion by the stupendous overthrow of Constantinople. Seven thousand janizaries mounted the breach, the last of the descendants of Constantine the Great died, sword in hand, upon the ramparts, and the thunder of his fall rolled with

* "Moral Sanitary Economy." By Henry M'Cormack, M.D., Consulting Physician to the Belfast General Hospital, &c. &c. "Education, Health, Order, Competence." Belfast, 1853, pp. 150.

reverberating peal throughout the extent of Christendom. The lapse of four hundred years has almost obliterated the remembrance of this startling fact. The modern period commenced; the discovery of America, the rounding of the Cape, and the application, if not the invention, of the art of printing, have transmuted the helmet, turban, scymitar, and cowl, into the untiring locomotive of a band of adventurers who are now exploring the globe with an intelligence, accuracy, perseverance, and intrepidity, of which there is not a similar example throughout the four thousand years that have preceded our own. It is an epoch without a parallel. Great Britain is becoming antiquated in the palmy hour of her supremacy. Grown grey in the service of feudalism, her serfs shake off their shackles, fly from her shores, and seek a fairer climate in a distant land. The white cliffs of Albion look down upon the daily emigration of her native population. Ireland is already, in part, deserted. The nation of merchandise, science, steam, naval and military prowess, is surprised in the midst of her coffers replete with bullion, and puzzled in the inmost recesses of her cabinet, so illustrious for its dignified line of astute and energetic statesmen. It is a conjuncture of affairs over which there is no control, and the issue of which no one can venture to predict.

The writers who dilate upon this singular theme would induce us to believe that no times had ever been so depraved as the present. But the present, in the short-sighted estimation of many, is always the worst, if it be not the best, because the unmanageable past is willingly discarded from our recollection, while the plastic future easily courts the imagination to do with it whatever it likes. For nothing can be easier than to build castles in the air for those yet unborn, to declaim against the corruption of the moderns, whose short-comings cannot be concealed, and to ignore the defects of our forefathers whose errors are lost in the darkness of ages. We forget the doom denounced against the great cities of antiquity ; or, if we do not forget their dismal reckonings, their actual enormities escape our sight in the shadowy length of the perspective. The great deluge and the cities of the plain belong to our schoolboy days ; and the separate counts of the indictment made out by the prophet in the open court of nations, now no more, against the haughty daughter of Babylon, are, we are pleased to fancy, curiosities reserved for the shelves of the learned, the museums of ancient marbles, and the terrible discourses of some young and enthusiastic divines. If we open Suetonius or Tacitus, or any of the authors of the *Historiæ Augustæ*, we read of morals so infernal and revolting, that they can proceed from none except the author of evil; but then with these we have nothing to do, because they are heathenish. And yet in Nero, Claudius, or Heliogabalus, we only perceive the extravagant exhibition of those very vices which constitute the elements of modern society, and which, in fact, the author of the essay placed at the head of this article has stepped forward to demonstrate with the pen of a moralist, at once keen, searching and uncompromising. His sight is microscopic, and the field of vision presented to our view, is like the scroll of the prophet, a parchment written within and without, and full of lamentation, and mourning, and woe. He appeals to facts for the truth of what he declares, as well as to the common experience of those who are moving in the midst of the scenes that now surround us. He asks whether civilization has checked the progress of immorality, and whether the mighty intellect of this country has succeeded in correcting the failures or miseries that tarnish the lustre of our name, and sap the vitals of our strength? He unhesitatingly answers himself in the negative. The infidel, ere now, has stepped forward in scorn, and has scoffingly asked, whether Christianity itself has not failed in its mission, and whether the awful scheme of Revelation has not lost its miraculous force of conviction, since millions are perpetually perishing without the knowledge of its truth, or else are living on, like the Pagans, in constant contempt of its commandments? The multitude are the creatures of circumstances, and the dupes of their passions, their hopes, and their fears, over which they profess to have no control. The world is a raft on the raging flood, covered with a shipwrecked crew, who are one after another being washed away, till the whole is swallowed up in the deep for ever. There is no image to represent the nothingness of life at all proportionate to its vivid reality.

In two short paragraphs we have exhausted the subject, and left nothing to be told except what is dull and hackneyed. The public have been so frequently regaled of late with accounts of the desperate wickedness lurking behind their doors, that they have lost their relish for carefully culled reports of horrors. For the present they have grown callous or indifferent. Even "Uncle Tom's Cabin," or the Stafford House committee of distinguished ladies, languishes, not from the want of any interesting materials, but because the subject is overdone or used up. It has relapsed into the monotonous routine of the day; and we are disposed to go on living as we have hitherto lived, insensible to the evils which do not immediately afflict ourselves. If courage is nothing more than the contempt engendered by a familiarity with danger, then we are certainly the most courageous people in the universe.

Only consider the catalogue of sins, both those of omission and commission, for which we are responsible, such as they have been drawn up by Dr. M'Cormack; the reader will perceive there is nothing in it which does not concern the community both individually and generally. Air, food, and clothing, are the sources or the means of disease and death, because, through ignorance, carelessness, or design, they are mismanaged and misunderstood. Marriage, and its opposite celibacy, are equally the disastrous springs of crime and misery. Employment, and the want of employment, are not less equally the destroyers both of the head and the heart, either from idleness, which is the father of mischief, or from unhealthy occupation, which is the foster-mother of pestilence, infirmity, and decay. The defects of education, both moral and intellectual, the ignorance of household economy, and the total disregard of physical training, are other still more direful modes by which, in early or later years, those who till the land or fill the cities, are brought down prematurely to the grave. The government of this country, so universally proposed as the model for all others, is at length discovered to be as bad as bad can be, in these respects. It has not only done nothing for the sake of ameliorating the condition of its subjects, but, what is worse, it is the direct cause of emigration, ignorance, pauperism, luxury, crime, starvation, infidelity, want, and woe. In short, if we are to credit almost every writer on this rueful topic, there is not an evil incidental to the lot of man which may not, in its last resource, be fairly traced up and imputed to the mal-administration of public affairs. It is this manner of treating the question that has rendered it, in the estimation of common sense, little else than a puerility ; and, as it usually happens when statements are overdrawn, the truths which they contain are disregarded in consequence of the illogical deductions of which they have, either wilfully or unintentionally, been made the handle. For the most part, people of the world turn a deaf ear to great enthusiasms; and history records nothing but cold comforts in the divine cause of charity.

"Numbers," says Dr. M'Cormack, "live in unnatural isolation or open profligacy. Individuals, whom no child is destined to call parent, swarm around." It is to the neglect of marriage, with its inextricable results, that he ascribes the moral degradation and physical wretchedness reigning among the masses of the population. One of the items that he points out in this dreary picture, is the important one of sheer ignorance on the part of women, whereby so many in every class of society are betrayed into consequences of which they could form no previous conception. We are inclined to demur to the plea of ignorance, and would rather substitute in its place the want of self-control. Be this as it may, they fall before they are aware of their danger, and, when once fallen, they not only never recover themselves, but remain in the bondage of sin, discarded, and left to perish by the wayside. There is no limit to the writers on this branch of the subject, British, American, and Continental, whose names are of the first order. In Prussia and Austria, the women do the work of their husbands and brothers, who lounge about in uniforms, and devour the produce realised by their more tender and less able-bodied drudges. Women are said to do the same in France, and other places, not omitting Ireland, and, until very recently, in the English and Scottish coal-pits, besides. The exalted female character is the last result of exalted civilization, the greatest rarity in the world. Another cause of female delinquency is occupation out of doors at night. Every one knows what the streets of towns are in these unseasonable hours. But necessity is imperious ; and, if it be true that of 1200 seamstresses only four had under-garments, there is but too obvious a reason for their providing themselves with clothing, whatever the means may be. The methods put in force to remedy evils so vast and crying as these, have hitherto proved quite inadequate to the purpose. Perhaps it is impossible to grapple with a calamity comprising the utmost destitution coupled with the utmost obliquity of the will. It sounds well to eulogize virtue, and to prescribe rules for propriety, discreet occupations, &c. But it is simple mockery in the face of those who stand alone in the wide world, who never knew the value of a good name, in the ordinary acceptation of the term, or are able practically to appreciate the word *home*, with all its soothing and endearing associations. It is not possible to heighten the colouring of sorrow so condign as this. The most detestable and most repulsive of all vice enables them to live, and without it they would starve. What a harrowing thought !

The want of employment is the prime source of crime and misery. The necessity of living presses upon us all. The excess of population, which ought to increase the occupation of hands in exact ratio to the increase of mouths, seems, on the contrary, to render the means of livelihood only so much the less. Every profession, trade, and craft is more than preoccupied with able competitors. Hitherto, pauperism has been on the increase. In Scotland, above 70,000 paupers are maintained, besides very large numbers in England and Ireland, most of whom, as the *Times* avers, if placed at the Antipodes, might secure comfort, if not affluence. The *work*houses are *idle* houses. Their inmates might be drilled to some employment, and forced to be productive. So might a larger number of the inmates of county lunatic asylums, barracks, and penitentiaries. Large tracts of waste lands are open for cultivation. There are in England and Scotland ten, in Ireland six, millions of acres ready to be reclaimed. Of the profit of so doing there can be no doubt. In 1818, the Dutch constrained beggars and vagrants, and even criminals, to labour and reform themselves, and the reward of their industry was the capability of their purchasing the soil they cultivated. But, in these kingdoms at least, and perhaps, also, in the States of Germany, the excess of population, which has led to pauperism, is at length finding a vent for itself in emigration. Considering the facilities of transit both by land and water, at the present day, we must expect such a popular impulse as nothing more than the natural consequence of such a state of things. It is a part of the breaking up of the old world and the repeopling of the new. No emigration has been recorded in history more extensive than this, and certainly not one so peaceable and intelligent. According to the last accounts, it amounts to about a thousand a-day from the various ports of the United Kingdom, for the past year. It might have been foreseen, though it could never have been provided against; and its effects on this country are beyond our calculations. There is no former period that we can measure with our own eventful era. The emigrants go to a land with a virgin and a golden soil, unencumbered and free from the exclusive privileges of caste; but then they have to contend with the difficulties of a new locality, clearing the ground, and locating the comforts of the civilized life they have just quitted. Under any circumstances, emigration is better than idleness, and the probability is that they send back to their mother-country either the produce of their industry, or gold in barter for the necessaries of life. And then the moral culture, so repeatedly insisted on by Dr. M'Cormack, will create itself, as soon as ever the individual finds himself independent, and bound to obtain his subsistence by means of a good character, conjoined with intelligence. It is one of those mental processes that cannot be forced, but which comes of its own accord. And thus the concert between the employers and the employed is a natural result of things cemented by mutual interests; it cannot be imposed, but must be left to itself, for wherever the interests are mutual, the concert will be unanimous.

The ragged children, who, sooner or later, if not brought within the sphere of educational influence, become petty thieves, ought to be protected by the state, and subjected to some industrial training. Count Rumford attempted this at Munich, and Robert Owen at New Lanark. Oberlin instituted infant schools at Waldbach, and Wilderspin organized schools for 27,000 children. The want of systematic education for the children of the forlorn and penniless is one of the crying evils of the times. At Weston-hill, Norwood, 900 children are reared in the ways of cleanliness, intelligence, and industry. The same might be done everywhere, and the good that would accrue to the community is incalculable; only something more permanent and substantial than the best individual zeal is clearly called for, in order to render undertakings of this kind effective and co-extensive with their laudable designs.

The next item of complaint on the black list, is that perplexing question which no one, in or out of parliament, has yet been able to answer satisfactorily to all parties—EDUCATION. Every one agrees that ignorance prevails to an alarming extent, and that its only corrective is education; but some maintain that it should be religious rather than secular, others secular rather than religious, and others, again, maintain, that the secular and religious should be conjoined. The real difficulty lies in religion, for every person or party of persons has his own views, and what this party or person thinks true, the other person or party thinks false. There is no end to the dispute, since there is no acknowledged umpire to settle the controversy. In the meanwhile, the unfortunate beings, who are the unconscious subjects of a ceaseless debate, grow up in the ignorance deplored by all, and fall short of the blessings which all the world purposes to bestow upon them. No one disputes the undeniable axiom, that every child, irrespective of sex, creed, or station, ought to be educated in the best possible manner. No one disputes this common-place truism, yet nothing is done to promote it upon an extensive and systematic scale, and the consequence is, the evil is progressive, and goes on multiplying, until it issues in its fatal results of crime and practical infidelity.

The arts and sciences alone have never yet exclusively civilized a nation, for they are the products, and not the causes of national superiority. Moreover, they concern the intellect rather than the morals. Unhappily, it must be owned that piety, virtue, and self-control, are not the constant attendants on learning and the splendid gifts of genius. Something more potent than mere *intellectual* culture is required to be put in force for the purpose of regulating the conduct of a responsible being, with a free will, like man, safely across the stormy ocean of life, from birth to death. The moral sciences alone touch the relationships of life. The intellectual is manifestly subordinate to the spiritual. The spiritual prepares the way for the intellectual, as the morning foreruns the day. Without the supernatural gift of faith, the mind is nothing but a hopeless chaos of scientific darkness, and moral impurity in continual conflict with itself.

Of the various kinds of education, the *home-education* takes the lead, and determines the future character of the offspring. This is the light in which we behold woman in her proper sphere of action. It is the noblest office that could have been devised for her. With the first dawn of thought, she determines the future destinies of mankind. In after years, it is impossible to obliterate the earliest impressions of our infancy. Tendencies, good, bad, or indifferent, have been implanted before we were conscious of our existence, and they have taken root so deeply that it is beyond all human force to tear them from within us. Our tutors may subsequently add something to our instruction, and we at last may do much for ourselves; but the starting point of our educational career, good or bad, rests exclusively with our mothers and nurses.

Household culture, above all, is the proper vocation of woman. The art of preparing wholesome food at the smallest possible expense belongs to no one, except the housewife. The teaching and the practice are hers. Those with small incomes must be content with rations, while those who are recognised as paupers, are forced to live almost upon nothing, or else upon such food as they can obtain, crude and ill-cooked, and therefore indigestible. Hence the gaunt forms and lean faces which prowl about the streets. Nevertheless, much of this pernicious state of things may be assigned to thorough ignorance of the culinary art. Most women, high and low, are ignorant of cookery, except in theory, if indeed so much as this. Ladies are entirely at the mercy of their cook, whose kitchen few can visit; while husbands in the lower and middling classes are at the mercy of their wives, who rarely know anything of the science of cookery, simply because they have never been taught. We might take a lesson in this respect from the savages. There are the pilafs of the Syrian desert, and the bill of fare among the Turks or Arabs is spoken of with rapture. A school for cookery—why not? or, as a previous part of the curriculum, a school for detecting adulterated food, which would be of the last importance.* In the Finchley-school, the girls wash, dry, and iron their clothes, which they bring home, together with a neatly written account. They also prepare a dinner weekly for their own use. At Belfast, the girls make, mend, sew, wash, &c., without omitting their general education at the same time. These are bright exceptions; for, from the Registrar-General's return, a large number of the women of England are unable to write their names at marriage—so uninstructed are the first instructors of mankind! In 1806, the state of the Duchy of Nassau, convinced of the evils of neglect, decreed that twice a week the female children of each several commune should assemble under a competent salaried matron for instruction in sewing, knitting, darning, washing, housekeeping—in short, order, economy, neatness, cleanliness, the first elements of civilization and refinement. This mode of education should be, if possible, pursued in the country, apart from the contaminated atmosphere and associations of a large town. The elastic spirit arising from the pure air of the country is of itself a powerful incentive to virtuous feelings and simplicity of mind. It must not be imagined that intellectual refinement grows up spontaneously, for, like everything else, it requires cultivating, and, if neglected, degenerates into gross vulgarity.

* The valuable series of papers published in our contemporary, the *Lancet*, under the head of "Analytical Sanitary Commission," are calculated effectually to put a stop to the cruel adulterations of both food and medicine which have been so unblushingly practised.

The section on criminal management is full of valuable information, but no conclusion is arrived at beyond that of the importance of early training. The poorer people have some excuse to offer for themselves; and yet poverty is only a relative term, and much of its dirt and wretchedness might be averted by prudence, foresight, and sobriety. Zealous individuals have at different times accomplished much towards the amelioration of the lower classes. The Philanthropic Society at Reigate is an instance of this, where the farm comprises 140 acres, and gives employment to 500 boys. At Aberdeen, Sheriff Watson originated an industrial school, where 400 youthful vagrants were housed and supplied with food, instruction, and work. Crime and ignorance are the most expensive commodities purchased by the community, and inflict a much larger drain on our incomes than is ever demanded on account of property or any other description of tax. They lie at the bottom of our heavy parochial dues.

The physical training so much practised by the ancients is more philosophic in its final results, than at first seems probable. As an evil mind renders the attitudes unconsciously gross, awkward, or ferocious, so the well-ordered movements of the body reflect a graceful pleasure to the mind, and evoke sentiments and emotions of a corresponding nature. But physical training must begin in the nursery, where alone it can be persisted in until it is formed into a habit. It requires wealth. It resides in the best quarters of the town, and the pleasantest localities in the country. It associates only with the best of its kind, and knows nothing of the ascetic, who has vowed voluntary poverty, and lives in the wilderness, whose raiment is sackcloth, and his food locusts and wild honey. On the contrary, it frequents kings' houses, and is so precious, that the gracefulness of a *danseuse* once cost the head of a saint. But, setting aside the licentious abuse of personal accomplishments, which always prevails in luxurious times, it is plainly manifest, that numberless maladies, both mental and bodily, proceed from the neglect of training the limbs, as well as the intellectual faculties, to the proper exercise of their functions. Witness the cavalry soldier who was once a clown, and the well-mannered youth who was once a clumsy boy. It constitutes the difference between the gentleman and the rustic, the savage and the civilized man.

Of food, which Dr. M'Cormac treats of, little need be said in this place, for medical literature is replete with essays on diet; nor of clothing, which is immediately concerned with the same subject. Different persons and classes of persons require different kinds of food, just as they do a different description of clothing. The lower classes are not clad warmly enough. Many children are lost from the want of it. Fashion modifies our attire, without respect to the necessity or reason of its use; and as to food, the fashion of the day is on the side of abstinence rather than on that of repletion. Hippocrates says, "They live the longest who live the best."

The concluding sections on air, drainage, and the prevention of disease, are chiefly stored with miscellaneous information relating to these subjects, to which we refer the reader; but we shall content ourselves with making a few interesting abstracts of those portions concerning mental hygiene, which is the specific object of this journal.

The number of lunatics in licensed houses in England, the metropolis included, is stated at 6731; in pauper asylums and hospitals, 8348, giving a total of 15,079. In 1814, the grand total was 20,000; while in that for 1847, the aggregate of the insane and imbecile, attendants inclusive, is reckoned at 30,000, an estimate supposed to be correct. In Ireland, there are said to be 15,000 insane persons, of whom 6113 only are under restraint. Inclusive of Scotland, then, we may assume the insane and imbecile in these islands to amount to 50,000, proportions which, if extended to the rest of Europe, constitute a serious aggregate. In the United States, the insane are also numerous, and, according to the "Journal of Insanity," Utica, 1846, the patients are very excitable. In Governor Hunt's message, it is stated, that in 1850, in New York State, there were 2506 lunatics, of whom 1106 were in asylums, and the rest in private families or poor houses. This frightful array of mental maladies must be the result of the destructive operations, hereditary or acquired, incessantly at work on the cerebro-spinal system. The numbers of either sex afflicted with insanity are about equal. Many, too sane for confinement, nevertheless continue to pursue their usual avocations with their intellects evidently debilitated and impaired. By the early addition of medical treatment, sixty per cent. of recoveries may be reckoned on.

Among the causes of insanity, the loss of self-control stands prominently forward. It is said, that every one could, by an effort of the mind, prevent the development of insanity. This affirmation is too sweeping; but in a certain sense it is correct. Hence the value of those prophylactic measures which tend to strengthen the mind and the body at the same time, and the importance of those pursuits which demonstrate the advantage of governing the appetites and passions, and rendering them submissive to the reason. The power of the will is a mighty one; and we believe there is no one who would not willingly resolve to preserve the just balance of his understanding, throughout life, if it were only pointed out to him in what manner he should exercise his will in order to do so. "In the battle of life," says Guizot, "in which we are called on to conquer, in the government of the inward man, in the midst of dangers and weaknesses, how many snares, enemies, victories and defeats, can be crowded into a day, an hour?" The evils of neglecting the proper cultivation of the mind are immense; and intemperance and insanity, the great curses of our civilization, act and re-act upon each other. The best writers on the disorders of the mind agree in assigning the first place to misguided morals, and show very clearly, that physical excesses are in reality nothing more than the evidence of deficient self-control. The difference between sanity and insanity depends simply on the degree of mastery over oneself attained to by each individual. In this light, we perceive loose morality degenerating into crime, and, at last, mingling in the twilight of guilt and innocence, so puzzling to the jurisconsults who in vain endeavour to draw the line of separation between them. Every maniac is not a criminal; but the criminal may have perpetrated his last fatal deed in a moment of mania engendered by self-will, depraved from his earliest years. Deficient in moral rectitude, both the physical and intellectual faculties suffer alike. Society, of course, can recognise nothing but the overt act, and the judge must pronounce sentence according to the verdict of "*guilty;*" but, beyond the court of justice, there is a humane feeling which acquits the convict, and seeks to recover the fallen wretch by gentler means than those of death, solitary confinement, or transportation for life. Vindictive punishments are the relics of a barbarous age, and disgrace the legislature of a period which affects to be so highly civilized and refined as our own. We live in hopes of seeing capital punishments done away with altogether, and buried in the same pit along with the bygone penal laws that once disfigured our statute-books. If the provisions of the ancient Roman law against crimes and offences of lese majesty were severe, their rigour was in accordance to the spirit of the penal system generally approved of in those days. In all ages there are predominant ideas, which are never called in question, and which, as it has been acutely remarked, must be laid to the charge of all mankind, or of none. Persons living in these days are shocked at their enormity; but the times are altered, and the more genial temper of Christianity appears to be pervading the world, and softening the asperities both of the governing and the governed.

Congenital idiotcy is not insusceptible of amelioration and relief. Dr. Güggenbuhl's successes in the Bernese Oberland are striking instances of this. His last report contains examples of great improvement in cretins effected by physical treatment. His establishment on the Abendberg shows how much may be accomplished by firmness, kindness, and intelligence judiciously conjoined. It is the same with confirmed insanity, which may be alleviated, if not cured, by education conducted on medical principles. Brierre de Boismont, in the *Annales d'Hygiène Public,* describes how sixty idiots, in Dr. Ferrus' school, at

Bicêtre, sang, danced, read, wrote from dictation, drew various figures, performed calculations, made shoes and furniture. At Colchester, the lame were made to walk, the dumb to speak, the helpless and imbecile to feed, dress, and keep themselves clean, while physical training, gardening, carpentering, shoemaking, reading, writing, singing, drawing, and music, were all taught, so far as the capacity of each individual would permit. At Bath, as at Highgate, every hour has its duties—order, kindness, obedience, love, are the only rules. The New York Asylum for Idiots has been attended with great success. In New England, kind treatment, proper food, and lively sports, with judicious discipline, have effected much good under the management of Mr. Richards. The humane conductors of these establishments are a pattern to others in the art of moral training. The management of the idiotic and insane requires persons of high moral and intellectual qualities. Each case must be treated according to its individual characteristics.

In the treatment of all nervous diseases, muscular labour in the open air is of late really much more attended to by psychologists. Fresh air is an anodyne, and fatigue in consequence of hard work is the best of opiates. For able-bodied lunatics, any employment is preferable to the stagnation of mind and body inevitably resulting from idleness. Every county report declares, that the employed are among the well disposed and quiet, the unemployed among the disturbing and disturbed portion of the population.

The worst kind of insanity, and even utter loss of mind, ensue from self-indulgence begun in early life. Parents have little conception of the nature of the evils they are engendering for their favourite children by giving way to their caprices and fancies, and fondly gratifying all their wishes.* It is a duty to deny them something for the sake of discipline, and to moderate their inclinations even for what is proper and good. A high character cannot be formed without its having been taught to endure long continued self-denial. The idiotic child is the evidence of moral perversion, if not the result of constitutional decay. Among the lower orders it is produced by the gin-palace, the factory, and the mine; among the more opulent it is fostered in the soft colliquations of affluence and ease. Vice and luxury, want and superabundance, are equally the sources of idiotcy, folly, extravagance, and crime. The reeking recesses of large towns send forth those pallid, joyless, stupid forms of human beings, which would make us start with horror, were not our gaze familiar with the sickening sight; while, along the parade, we are so habituated to the wasted features of

* Plato ascribes the fall of the Persian Empire to the over indulgence of their children: in other words, spoilt children ruined a kingdom.

dissipation and fashion, that we mistake them for the standard of beauty, and actually forget that they are nothing more than spectres of mental deformity and bodily degeneration and abuse. Of such as these are the inmates of lunatic asylums, and the public are little aware that the cost of insanity treads hard on the outlay for crime. Colney Hatch, for example, a recent erection, and but one out of many, has already entailed an expenditure of 300,000*l.*!

A careful education in early life, which would instil a just appreciation of the world and its allurements, would save many from ending their days, or blighting their reputations by being forced to reside for some length of time, in the lunatic asylum; and doubtless, it has preserved many from the worst of human calamities, the loss of reason. And yet we venture to repeat that education, even among the wealthy classes, is miserably neglected. A smattering of learning is obtained at petty boarding schools, where the teacher requires teaching, and the pupils are taught nothing that concerns the moral grandeur, the end and aim of an immortal being. Consequently, they enter the world practically ignorant of their chief duties, and are considered sufficiently accomplished if they are acquainted with the received modes of society, and are able to speak on the current topics of the day without embarrassment or conceit. But this is not education in its loftiest sense—it is only ease and fluency learnt by rote,—phraseology fixed in the memory without informing the understanding; and the end of it is, indiscretion, debauchery, and criminal engagements, a seared conscience, and the sting of remorse without the power of reforming themselves. The Christian principle is as little discernible as it was in the education of the Roman youth; who were passed over to the care of Greek pedants, whose interest it was to corrupt the passions of those whom they wished to flatter, and whose office was despised by the Romans because they purchased it at any price they pleased. Tutors of this description imparted a frivolous taste for literature, false politeness of manners, a love for the theatre, shows and dances, a low taste for jockeyism, intrigue, and ostentation. By these means they infected the national morals in their very springs of action, and scaled the citadel of virtue by entering through the breach already opened by the philosophy of Epicurus, and but imperfectly repaired or defended by the doctrine of the Stoics. Tacitus, who describes the excessive luxury of Rome, gives a very able speech of the Emperor Tiberius, animadverting upon it. Apicius, who had squandered his enormous wealth and was reduced to only ten millions of sesterces (100,000*l.*), committed suicide from his dreadful sense of poverty. Tacitus calls him *dives et prodigus*, and imputes to him the worst of crimes. Contrasted with this dereliction of morals and disgusting barbarity in the midst of refinement, stand out, in bold relief, such characters as Cicero, Sallust, Pliny, Tacitus, &c., who shone so brightly on account of their splendid education; and what would such men not have been, had they only been Christians? For it was not with them as it is with us. The moderns isolate their sciences too much, and fall into the same error in this respect as the Hindoos and Egyptians, with their castes. The soldier knows nothing of law, nor the lawyer of military tactics. The civil laws are separate from the administration of affairs. Religion has its proper clergy, and finance its chancellor of the exchequer. Politics, diplomacy, merchandise, the learned professions, are confided to separate hands, and distributed differently. But among the ancients during the republic and the empire, the same man was at the same time a warrior, an advocate, a magistrate, a judge, a financier, a pontiff: nothing was strange to him. For several years he had served in the field and was intimate with the qualities of the soldier, and the operations of an army during an active campaign. He had sat in the senate for twenty years, had listened to every debate, and was intimate with the state of public affairs, of the most important and critical nature; and, after having passed through and discharged the functions of every office in the republic, his knowledge and experience were reckoned to be at last complete, fit for the public service, an honour worthy of his name, and a distinguished ornament as well as the support of the Roman fabric. There may be many excellent reasons why an education of this description could not be carried into execution among ourselves, and very just motives may be alleged for the modern distribution of politics, arts, and sciences among persons who profess each one in particular; nor have we brought forward this old *régime* with any view of enforcing it on the attention of our contemporaries, but only for the sake of showing what our education is *not* in the present day, and what it might be made with every possible advantage to ourselves and those who follow us. Be this as it may, one thing is clear, that the general ignorance prevalent throughout the community is a positive evil, fraught with the most disastrous consequences, and such as will, sooner or later, force itself upon our notice in a manner not to be disregarded. The abundance of books published is no proof of solid information. It only proves the universal appetite for knowledge, and the greater necessity there is for directing it aright, and supplying it with wholesome nutriment.

We are not among those who look with a desponding eye on the epoch in which we are living, but neither have we any very sanguine expectations of an approaching millennium: our views are much more sober. We perceive that the bulk of mankind is rapidly improving in every respect. The exclusive privilege of rank and title unsupported by real personal merit, is almost at its last gasp. It cannot survive much longer. The means of livelihood are increasing, while the gifts of fortune are changing hands. The old nations of Europe have all of a sudden grown extremely old. Their institutions are wearing out, and must be repaired, altered, and adapted to a new condition of things, in which the intellect will play a higher part than hereditary greatness, the pen will be sharper than the sword, and virtue of more real value than brute force and extensive armaments. There may be some partial interruptions to this progress of events, but the issue of them is certain, and is not far off. As journalists, it is our part to study the mind in its various phases, both individually and nationally, to point out the various mental phenomena as they arise, and to lend our voice in assisting the improvement of the mind and morals for the public good.

That we are not far from the truth in our surmises, a glance at the social revolutions of the last three hundred years will quickly assure us. The establishment of the European colonies is a fact exclusively modern. The mind of the foregoing periods had never conceived so vast a design, nor had it the means of executing its projects, even if it had been equal to the idea of them. The "people," as they are emphatically styled, were still in their serfdom, and their intelligence was fettered together with their personal liberty. As soon, however, as Europe felt herself disembarrassed of her inconvenient fetters, the highest destinies expanded before her. Unlike the crusades against the Ottoman power in the East, a new crusade, of a very different character indeed, was undertaken towards the West. It was mean and paltry in its apparatus when compared with the glittering equipments displayed by the knights of chivalry, but it was altogether grander and more imposing in its efforts and results. Millions of soldiery were called upon to go and rescue the Holy Land, which was lost as soon as won; but a few thousand Spaniards and Portuguese were alone sufficient for the conquest of America on the one side, and India on the other. Mighty as the achievement has proved itself to be, it was but as child's play in comparison with the futile armaments of the middle ages. The Europeans laughed as they consigned the universal dominion of the New World to the hands of a few necessitous adventurers; for the famous conquerors of Hindostan, Mexico, and Peru were little else than the offcasts of society. Columbus was worth nothing but his genius; and if we consider the infamous manner in which the great trading companies first recruited their armies, we must acknowledge, that the far-off lands lying beyond the Atlantic and the Indian Ocean surrendered themselves at discretion to some of the most desperate and worthless of the restless European populations.* This may alone suffice to account for the excesses and enormities that sullied the first occupation of the two Indies (East and West), besides the inevitable consequences of so many reckless vagabonds, who suddenly found themselves alone and destitute in the midst of a feeble-minded race whom they resolved to subdue by terror instead of kindness. Against the Spaniards, indeed, the worst of accusations are denounced. The Europeans are regarded as ferocious and blood-thirsty. Perhaps, the entire history of modern and medieval Europe does not acquit them. A century has not passed since the first French revolution, and our own country has not been less sanguinary in its continental, eastern, and domestic wars. But run through the ancient forests of the New World, now forming the territory of the United States, and see whether the Anglo-Saxon race in America, scarcely yet two centuries old, has not done wonders in redeeming its character for peace, and in making amends the most honourable for the savage cupidity of a few heartless soldiers, at their first landing (the buccaneers, flibustiers, &c.) Asia has not fared so well as America in this respect. India alone has retained with amazing obstinacy her manners and laws, the growth of centuries. But we need not despair. It may require a long time before the liberal spirit of the present age shall have thoroughly penetrated the compact masses of the Hindoo people, hardened into a concrete by the operation of countless generations of a uniform character and aspect, coeval with the earliest legends of mankind. It is not probable that the mere sentiment of personal independence and social unity should be able to overthrow barriers at once so ancient and consolidated as these prejudices of the imperturbable Hindoo. Moreover, we must bear in mind, that merchants are not either moral or religious missionaries. The warehouse,

* Jusque dans les projets de colonisation formés officiellement, nous retrouvons la même composition. Si l'on fait des envois d'hommes au Nouveau-Monde, ce sont toujours des malfaiteurs qu'on tire des prisons ou des galères; les cargaisons de femmes se composent toutes de filles publiques; et quand le vénérable Las Casas ne veut que des honnêtes gens pour sa colonie, il n'en trouve pas deux cents dans toute l'Espagne qui consentent à le suivre. Nous ne parlons pas de Botany Bay.—*Histoire Moderne.* Paris, 1843.

England, indeed, has hitherto had her colonies to receive, against their will, her expatriated criminals. But, as we observed the other day, and now with the greatest satisfaction repeat, transportation to Van Diemen's Land and other Australian colonies is to cease altogether, while even to Western Australia it can only be continued for a short time, and to a very limited extent. Nor can it be other than a gratifying contrast to reflect upon, that while the legislature is and will be compelled to consider the subject of secondary punishments for confirmed criminals, they (the Reformatory Institution at Birmingham) are actively engaged in providing the best instruments and employing the most efficacious agencies for dispensing with such punishments, by affording the youthful, and as yet yielding criminal, the means of reformation, and the opportunity of prosecuting an honest, industrious, and useful career.—*The Times*, Feb. 24, 1853.

the banker's ledger, and a bill of exchange, are the worst of preachers before a philosophic sect, such as a Brahmin or a Buddhist. The pantheism of the East may obey, but it can neither love nor understand, the monetary system nor the military despotism of a host of foreign mercenaries. More expanded ideas are, however, gaining ground. "The Emperor of the Afghans," says M. Chardin (*Histoire des Colonies Européennes aux Indes—Bibliothèque Populaire*), "has recently received a letter written in French by an Indian rajah. The Rajah of Tanjore, a stipendiary of the English, understands modern chemistry, reads Shakspeare, and possesses a portrait of the first Napoleon." A revolution is slowly at work in the gorgeous East,—the dark races are gradually being carried away in the torrent of progressive improvement; the European mind is spreading over the entire surface of the earth, the hour of intellectual triumph is at hand, and perhaps it is nearer than we now suppose. If the few clever clerks in Leadenhall-street, who preside over the destinies, should happen to fail in the government of our Anglo-Indian empire, their extensive rule would fall to pieces; possibly the Russian troops might step forward and take it in hand; but certainly the modern descendants of Ham would have seen their last day, and a new order of things would arise upon the ashes of primeval Asia.

In America this revolution is finally settled. The new world has affranchised itself. Oceania, New Zealand, and Australia, are doing the same. They are severing, or beginning to sever, themselves, from the agents of their fatherland. Nationality is dying a natural death. European in their language, their laws, their manners, their literature, and their tastes, they boldly assert their claim to independence, and avow their intention of beginning from henceforth a career of fortune of their own. They disdain the rule, because they are conscious of being equal to the powers of Europe. America has led the way in this course of events. From a dependency she has, in less than a century, grown up into a mighty nation, and stands forth as the outpost and advanced guard of European civilization.*

In the psychology which we have, from the commencement of this journal, avowedly undertaken to investigate and dissect, we have at no

* The subversion of twenty empires is the price at which Providence has accorded to Europe the light of modern civilization.—*Abel Rémusat.* What the price shall be at which the southern hemisphere will surpass the nations of the Old World, is a treasure laid up in store for the pen of some future historian, whose glorious task it shall be to record the destinies of New Zealand, Australia, or the penal settlement of Botany Bay. A portion of land, more than equal to the whole of Europe, is yet unchronicled in the list of nations. Its extent, its climate, and the riches of its alluvial soil, are already attracting crowds from the northern hemisphere; they bring with them the resources, the science, and the refinements of the experience of ages; and its population has already begun at a point of elevation equal to our own.

time shrunk from demonstrating its operations in the totality of mankind as well as in the person of each individual. The history of mind is that of man for six thousand years. We behold him, like a child under the tuition of a master, being instructed step by step, partly by precept and partly by experience, until he has reached the age of manhood, when he can at length exercise his faculties, and judge and act for himself. We apprehend that the present is an age of comparative maturity, such as has not yet been witnessed before. That this notion is not chimerical on our part, nor the boasting of a petty vanity which delights in extolling its own times and nation, is unanswerably proved by the increasing order, discipline, and intelligence of the masses of the community; and even the complaints that are made respecting the want of education and the alarming prevalence of crime, do not so much show the actual existence of these evils, as they imply the earnest desire on all sides for adopting the proper means for the improvement both of the mind and morals. Were it otherwise, it is not likely the lower orders would listen so patiently as they do to the constant upbraidings and reproofs repeatedly uttered in their hearing, the plans suggested for their correction, or the means propounded for their amendment. The truth is, they enter into these views as heartily as we do ourselves. They are solicitous for their own advancement, and willing to make every effort for the purpose of elevating themselves and their offspring in their own esteem as well as in that of others. It is but natural they should do so. Why should we marvel at it? The only wonder is, that this popular movement has been delayed to so late a period of the Christian era, nay, almost entirely until within the memory of the present generation; and that, after a lapse of eighteen hundred years, we should be only just beginning to open our eyes to the probability that perhaps even the *Peace Society* may not be so egregiously mistaken as their opponents would represent them to be. For our part, we own that we hail it as the harbinger of better things yet to come, and that the approach of a wiser spirit among the governed and the governing is not far off. One thing is decided, that brute force is fast falling in the general estimation of mankind, and that it is less requisite in just proportion to the higher intelligence of all. The pamphlet of Dr. M'Cormac has given rise to this train of thoughts in which we have indulged ourselves. We have spoken according to our brief. If our pleading has been special, it has been in favour of the commonwealth of virtue, the noblest cause there is, as well as in direct support of those grand principles of progress and intelligence, of which no one need blush to avow himself the advocate, nor hold himself aloof from too sensitive a fear of being pointed out as their follower and abettor. Mere novelty is puerile and contemptible; but obstinacy argues stupidity or ignorance. Our part will not suffer us to lie in the rear, but dictates to us a line of duty in the elucidation of the mental phenomena, from which we cannot swerve, and in the ample discharge of which consist our exercise and delight.

---

### Art. III.—HAYDON ;—A PSYCHOLOGICAL STUDY.*

